# Pseudoaneurysm of the Pancreaticoduodenal Artery Associated with Duodenal Diverticulitis

**DOI:** 10.1155/2019/2831234

**Published:** 2019-07-22

**Authors:** Scarlett B. Hao, Dale B. Johnson, Hugo J. R. Bonatti

**Affiliations:** ^1^University of Maryland Shore Regional at Easton, MD, USA; ^2^Vidant Medical Center, Greenville NC, USA; ^3^Meritus Surgical Specialists, Hagerstown MD, USA

## Abstract

**Background:**

Duodenal diverticula tend to be asymptomatic; however, patients may develop duodenal diverticulitis.

**Case Presentation:**

A 66-year-old Caucasian man presented to our emergency room with a two-day history of right-sided abdominal pain, chills, tachycardia, nausea, and emesis. His WBC, lactic acid, and bilirubin were elevated. CT-scan revealed an inflammatory process involving the gallbladder, the duodenum and ascending colon, a mesenteric soft tissue mass, and a diverticulum of the second portion of the duodenum. He was admitted, antibiotics were started, and he improved clinically over the next 36 hours. Repeat triple contrast CT-scan showed a two cm pseudoaneurysm (PA) of the pancreaticoduodenal artery causing a mesenteric hematoma. The inflammatory changes had significantly improved, and WBC and CRP were normalizing. Repeat CT-scan three days later demonstrated an interval increase in size of the PA. Angiography through celiac access and gastroduodenal artery demonstrated predominant inflow to the PA from the inferior pancreaticoduodenal artery. The superior mesenteric artery was accessed showing a replaced right hepatic artery hindering access to the branch feeding the PA. The patient was transferred to a specialized facility where ultimately occlusion of the PA inflow was obtained. The patient recovered without any complication from this rare condition.

**Conclusion:**

This seems to be the first reported case of duodenal diverticulitis causing a PA of the pancreaticoduodenal artery. Antibiotic therapy together with percutaneous embolization of the bleeding source resulted in a good outcome.

## 1. Introduction

Duodenal diverticula or sac-like protrusions of the bowel wall in the duodenum are noted in up to 27% of patients receiving endoscopy and up to 23% of patients at autopsy [1–3]. The majority are asymptomatic or are associated with vague gastrointestinal symptoms including postprandial pain and bloating [4]. The most common type of duodenal diverticula occurs juxtapapillary along the second portion of the duodenum. Rarely, diverticula may be involved in pancreaticobiliary disease, such as in Lemmel's syndrome where an impacted enterolith in the duodenal diverticulum obstructs biliary outflow [5]. Duodenal diverticula and pancreaticobiliary disease may or may not have a causal relationship [6–8].

Duodenal diverticulitis and perforated duodenal diverticulum are rare conditions, and patients present with acute abdominal pain, nausea, fever, and elevated WBC [3]. The condition may be associated with hemorrhage due to erosion of a blood vessel at the base of the diverticulum [9, 10]. Management of duodenal diverticular pathology varies based on the context, the presence of complicating factors, and comorbid pathologies. In a recent article, Thorson et al. summarized a total of 162 cases of perforated diverticulum and emphasized that diagnosis has dramatically improved after 1989 with the use of CT-scan. While surgical intervention was commonly done in older publications, it is accepted today that in most cases, the condition will respond to antibiotic therapy and bowel rest [9].

Hemorrhage from the pancreaticoduodenal artery and its branches has been found after trauma and surgery and in patients with acute and chronic pancreatitis amongst other causes [11, 12]. Pseudoaneurysm formation may be observed, which represents a potentially life-threatening condition and warrants intervention. Surgery may be necessary in some cases; however, interventional radiology offers a less invasive approach [11]. It should be considered that inflow to the pancreaticoduodenal arterial arcade may be from the celiac trunk through common hepatic and gastroduodenal artery or through a branch of the superior mesenteric artery, and access to this vessel may be difficult.

We report on a 66-year-old Caucasian man with acute right-sided abdominal pain associated with duodenal diverticulitis who developed a pseudoaneurysm of the pancreaticoduodenal artery. He was successfully treated with antibiotics and endovascular therapy.

## 2. Case Report

**Figure 1:**
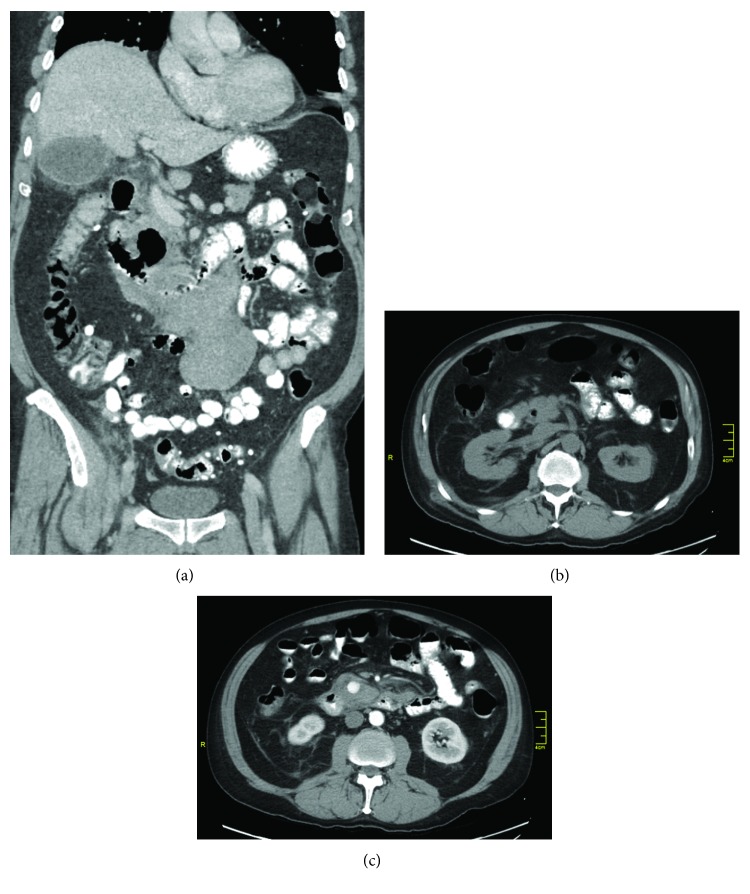
(a–c) CT-scan: (a) large duodenal diverticulum (arrow), mesenteric hematoma, stranding of surrounding tissue. (b) Contrast in diverticulum (arrow). (c) Two cm pseudoaneurysm with hematoma (arrow).

**Figure 2:**
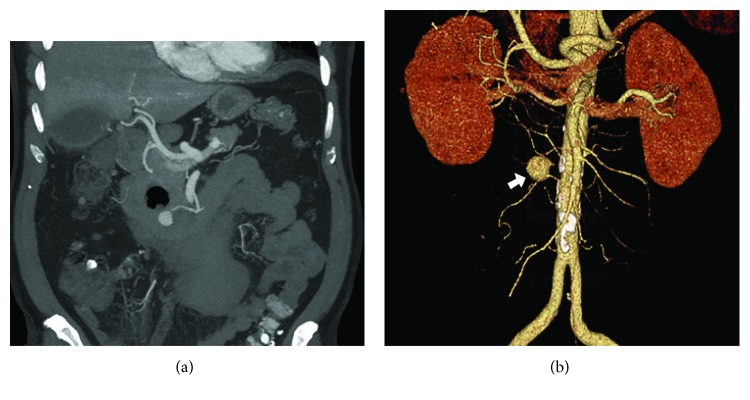
(a) Follow-up CT-scan: improved right upper quadrant inflammation, duodenal diverticulum (arrow), expanding pseudoaneurysm (arrow). (b) Angiography reconstruction: large pseudoaneurysm (arrow) of the pancreaticoduodenal artery.

A 66-year-old Caucasian man with a past medical history of gastroesophageal reflux presented with two days of right-sided abdominal pain, chills, nausea, and emesis. He was tachycardic but afebrile and had significant right upper quadrant (RUQ) tenderness. Blood work revealed leukocytosis, an elevated lactate of 3.7, and elevated bilirubin of 2.0. CT-scan revealed gallbladder wall thickening and pericholecystic fluid, wall thickening and fat stranding of the duodenum and ascending colon, and a mesenteric soft tissue mass. He was resuscitated, placed on bowel rest, and started on intravenous ertapenem at a dose of 1 g daily. Over the next 36 hours, clinical improvement was observed and WBC and CRP started to normalize. Repeat CT-scan with oral and intravenous contrast showed improvement of the RUQ inflammatory process and revealed a diverticulum of the second portion of the duodenum-associated inflammatory changes and a large pseudoaneurysm of the inferior pancreaticoduodenal artery. The scan also delineated the previously noted soft tissue mass as a stable hematoma (Figures 1(a)–1(c)). Gastroenterology was consulted with a plan to perform endoscopic evaluation after clinical improvement and resolution of the hemorrhage. The patient further improved clinically and was discharged on a clear liquid diet and oral antibiotics. Repeat scan three days later demonstrated an interval increase in size of the pseudoaneurysm (Figure 2). He was asymptomatic, but due to the growth of the lesion, interventional radiology was consulted. During angiography, the celiac access was cannulated and the gastroduodenal artery was reached. Selective angiography demonstrated predominant inflow to the pseudoaneurysm from the inferior pancreaticoduodenal artery. Therefore, the superior mesenteric artery was cannulated showing a replaced right hepatic artery. This artery hindered access to the branch feeding the pseudoaneurysm, and on multiple attempts, the guidewire could not be advanced. There was no active hemorrhage, and the patient remained hemodynamically stable. He was transferred to a specialized interventional radiology facility. On a first attempt again, the inflow to the pseudoaneurysm could not be accessed; however, during a second attempt two days later, occlusion of the pseudoaneurysm inflow was obtained. The patient recovered without any complication and was well at his six months of follow-up. He had no further episodes of duodenal diverticulitis. Interval endoscopy confirmed the presence of a large duodenal diverticulum without inflammation.

## 3. Discussion

While studies have shown an association between juxtapapillary duodenal diverticula and both primary and recurrent bile duct stones, the mechanisms behind the association between pancreaticobiliary disease and duodenal diverticula are varied and not without debate [6, 7]. Increased bacteriobilia, sphincter of Oddi dysfunction, and impaction leading to mechanical obstruction as potential mechanisms of pancreaticobiliary disease secondary to duodenal diverticula are well described [13]. Duodenal diverticula, similar to the more common colonic diverticula, can be complicated by diverticulitis and even perforation, resulting in RUQ pain that may be mistaken for gallbladder pathology. Chen et al. described a classification system and noted complications tended to occur more frequently in cases of the ampulla opening directly into the diverticulum [6].

**Figure 3:**
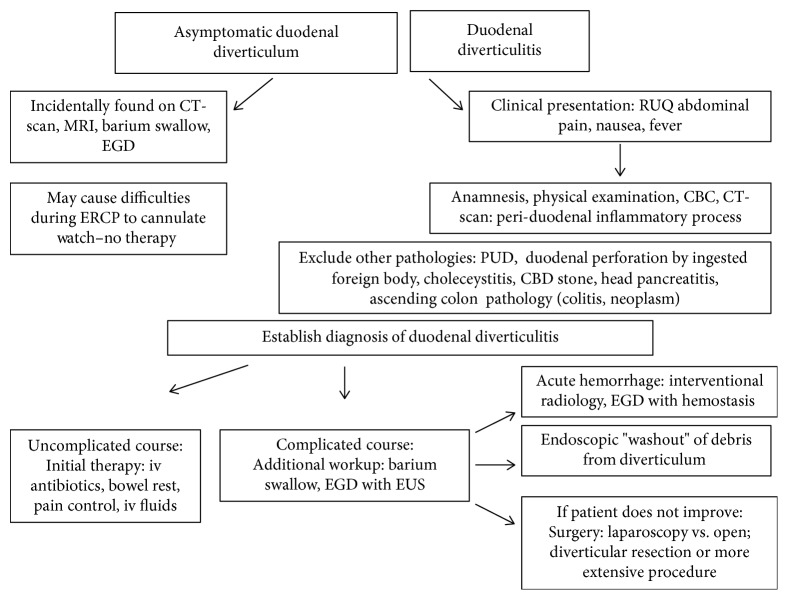
Algorithm on how to diagnose and manage duodenal diverticulosis and diverticulitis.

Given its proximity to other anatomic sites more likely responsible for symptoms, duodenal diverticular disease may be difficult to diagnose without several imaging modalities. Schroeder et al. reported anecdotes of duodenal diverticulum misidentified on initial imaging as perforated duodenal ulcer, pancreatic cyst, or potential malignant soft tissue mass causing mass effect [2]. Better imaging using CT-scan seems to be largely contributing to the improved outcome of the disease in recent years and a decline in surgical interventions [9]. Nevertheless, a multitude of pathologies of organs in close proximity to the duodenum must be considered before final diagnosis of the rare disorder can be established. Whereas incidental finding of a duodenal diverticulum is not uncommon during EGD, endoscopy does not play a major role in diagnosing acute duodenal diverticulitis. Endoscopic washout of debris from the diverticulum has been suggested and may promote healing [14].  Figure 3 shows an algorithm on how duodenal diverticulosis and diverticulitis may be diagnosed and managed.

With respect to treatment, asymptomatic diverticula are generally left alone and bowel rest together with antibiotic therapy is now widely accepted as primary treatment of uncomplicated duodenal diverticulitis similar to the management of uncomplicated colonic diverticulitis [15]. In cases of concomitant pancreaticobiliary disease with duodenal diverticula, treatment of both pathologies is important to reduce recurrence. Stone removal may be necessary, but difficulty with cannulation during ERCP in the presence of a duodenal diverticulum has been reported [16].

Surgical management has historically included approaches such as diverticulectomy and cholecystectomy, choledochoduodenostomy, distal gastrectomy combined with gastrojejunostomy, and duodenojejunostomy. The offered surgical procedures may be excisional and diverting. Due to the variation in specific location of juxtapapillary duodenal diverticula and its proximity to critical structures, diverticulectomy has a risk for significant morbidity and mortality; by comparison, diversion has been shown to be safer and simpler [7].

In our case, a surgical approach was considered; however, a nonoperative management was chosen. The diverticulitis responded quickly to our therapy confirming data from the literature; however, the large and expanding pseudoaneurysm mandated interventional therapy. Occlusion of the bleeding source turned out difficult due to inflow from the SMA and the presence of an accessory right hepatic artery but was ultimately successful.

To summarize, duodenal diverticulitis is a rare disease and an association with a pseudoaneurysm of the pancreaticoduodenal artery has not been reported thus far. Antibiotics and interventional radiology were able to control the disease.

## Abbreviations

EGD: Esophagogastroduodenoscopy
ERCP: Endoscopic retrograde cholangiopancreatography
MRI: Magnetic resonance imaging
CT-scan: Computed tomography
PUD: Peptic ulcer disease.
